# Decoding the molecular mechanism of parthenocarpy in *Musa* spp. through protein–protein interaction network

**DOI:** 10.1038/s41598-021-93661-3

**Published:** 2021-07-16

**Authors:** Suthanthiram Backiyarani, Rajendran Sasikala, Simeon Sharmiladevi, Subbaraya Uma

**Affiliations:** ICAR-National Research Centre for Banana, Thogamalai Road, Thayanur Post, Tiruchirapalli, Tamil Nadu 620 102 India

**Keywords:** Computational models, Computational biology and bioinformatics, Plant sciences, Plant development

## Abstract

Banana, one of the most important staple fruit among global consumers is highly sterile owing to natural parthenocarpy. Identification of genetic factors responsible for parthenocarpy would facilitate the conventional breeders to improve the seeded accessions. We have constructed Protein–protein interaction (PPI) network through mining differentially expressed genes and the genes used for transgenic studies with respect to parthenocarpy. Based on the topological and pathway enrichment analysis of proteins in PPI network, 12 candidate genes were shortlisted. By further validating these candidate genes in seeded and seedless accession of *Musa* spp. we put forward *MaAGL8, MaMADS16*, *MaGH3.8*, *MaMADS29, MaRGA1*, *MaEXPA1*, *MaGID1C*, *MaHK2* and *MaBAM1* as possible target genes in the study of natural parthenocarpy. In contrary, expression profile of *MaACLB-2* and *MaZEP* is anticipated to highlight the difference in artificially induced and natural parthenocarpy. By exploring the PPI of validated genes from the network, we postulated a putative pathway that bring insights into the significance of cytokinin mediated CLAVATA(*CLV*)–WUSHEL(*WUS*) signaling pathway in addition to gibberellin mediated auxin signaling in parthenocarpy. Our analysis is the first attempt to identify candidate genes and to hypothesize a putative mechanism that bridges the gaps in understanding natural parthenocarpy through PPI network.

## Introduction

The term parthenocarpy refers to ovary developing into a seedless fruit in the absence of union of female and male gametes. It has been reviewed in large number of horticultural crops such as grape, tomato, mandarins, banana, opuntia, pepino, eggplant, cucumber and capsicum^1^ and stated that parthenocarpy can be achieved as a result of over expression of endogenous hormones in the ovary^2^ and can be genetically controlled^3,4^. From the inheritance pattern of parthenocarpy in various crops, it has been reported that the trait parthenocarpy is governed by a single dominant gene in eggplant^5,6^, single recessive gene like in *Capsicum annum*^7^, more than two recessive genes in tomato^8,9^, a single dominant gene in pepino^10^, a single incompatible dominant gene in cucumber^11,12^ and two major additive, dominant-epistatic genes in cucumber^13^. Phytohormones such as auxin and GA (Gibberellin) playing predominant roles in parthenocarpic fruit development such as tomato^14^, *Arabidopsis*^15,16^, apple^17^ etc., It is also being commercially exploited in horticulture crops^18^ through exogenous use of irradiated pollen, natural or synthetic hormones such as auxin, GA, IAA etc., during ovary development^19–21^. In spite of so many reports, the molecular mechanism involved in natural parthenocarpic fruit development is still unclear and candidate genes for the trait parthenocarpy have not been identified till date. To understand the molecular mechanism involved in parthenocarpic fruit development, comparative transcriptome analysis has been studied between parthenocarpic and non-parthenocarpic (seeded) accessions in many horticultural crops such as eggplant^22^, citrus^23^, litchi^24^, oil palm^25^ etc. Many researchers have tried to identify the parthenocarpic mechanism by studying the expression profile of induced parthenocarpic fruit either via exogenous application or through mutation or genetic transformation^26,27^.

Among the horticulture crops, banana an economically important crop but its seediness hinders its improvement through conventional breeding approach. Unlike in other crops, ploidy status, intra and inter specific hybridity nature of commercial cultivars/varieties have led to chromosomal imbalance during gamete formation that plays a determinant role in seedless fruit formation. Only limited studies are available for understanding the genetics of parthenocarpy in banana and plantains. It has been stated that the trait parthenocarpy in banana is governed by three independent complementary genes in which the absence of even one dominant gene that resulted in seediness^28^. Similarly, based on the segregating pattern, it has also substantiated that parthenocarpy is governed by three genes^29^. Further, it has been postulated that among the ancestor genome (A and B) of the present day commercial cultivars, “A” genome coming from *Musa acuminata* (AA) contributes to the female sterility resulting in vegetative parthenocarpy^29–31^. However the loci or the genetic factors responsible for the trait parthenocarpy are not yet identified because of their inherent nature like male and or female sterility, heterozygous nature of parents, unreduced gamete formation etc.

**Figure 1:**
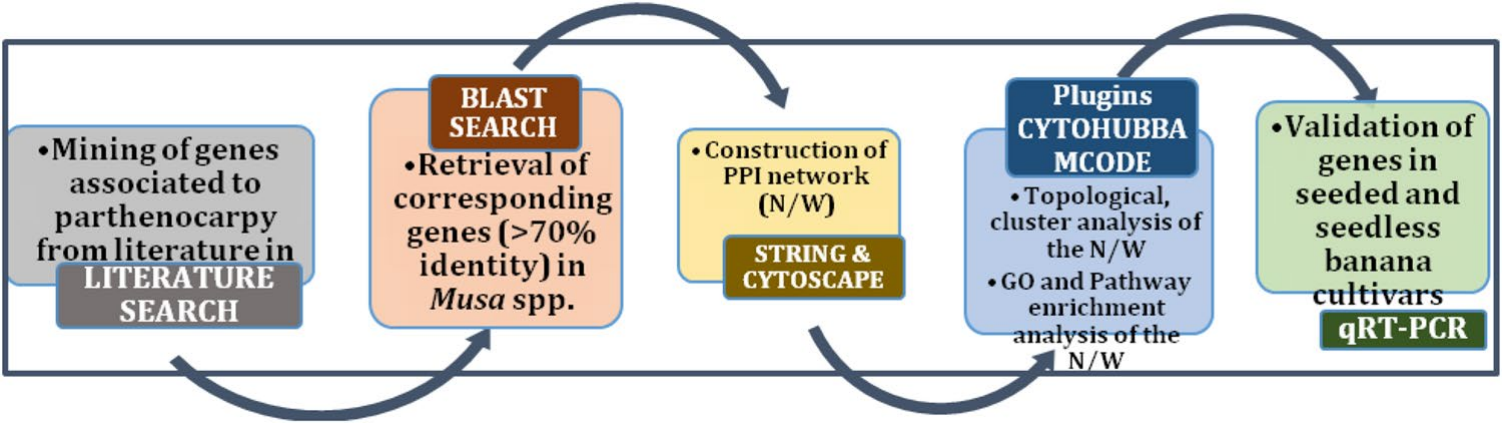
General workflow of the study. In 1st stage, genes associated with parthenocarpy were mined through literature search. In 2nd stage, corresponding orthologous genes in banana were retrieved using BLAST. In 3rd stage, PPI network was constructed using STRING and Cytoscape software. In 4th stage, topological, cluster analysis identified candidate genes and proposed putative pathway. In 5th stage, candidate genes were validated in *Musa* spp. through qRT-PCR.

The lack of availability of data associated to seeded and seedless accessions of *Musa* spp. hampered perceiving the knowledge on genetic mechanism/factors involved in parthenocarpy. In such scenario, “omics” information related to parthenocarpic trait of various species which are hugely deposited in public databases could be exploited through computational approaches. Several in-silico methods such as sequence similarity, evolutionary relationship, detection of SNPs, high throughput gene expression analysis and protein–protein interactions (PPI) etc., could be applied for identifying the genetic factors responsible for parthenocarpy in *Musa* spp. Of which computational prediction of PPI from the gene expression profiles has been widely implemented for the prediction of candidate genes that regulate any complex trait^32^. Hence in this study we focused on “proteogenomics” approach by mining the differentially expressed genes (DEGs) of seeded and artificially induced parthenocarpic fruits of various crops, tomato, eggplant, capsicum, grapes, citrus, apple etc., for the identification of candidate genes responsible for parthenocarpy in *Musa* spp. The Graphical abstract of the work flow used in the current study is shown in Fig. 1. Genetic factors from various orthologous species involved in the parthenocarpic fruit formation and their respective homologous genes in *Musa* spp. were taken for the construction of PPI network for the trait parthenocarpy, since it is evidenced that PPIs are conserved in different orthologous species^33^. The shortlisted genes were validated in seeded and seedless accessions of banana to identify the candidate genes for natural parthenocarpy in banana.

## Results

### Construction of parthenocarpy associated PPI network

A total of 210 DEGs were extracted from the transcriptome profile of various crops with respect to artificially induced parthenocarpy and from the genetically modified crops. BLAST analysis of these genes against *Musa* spp. (https://banana-genome-hub.southgreen.fr/blast) displayed a hit with 156 orthologous protein sequences with an identity of ≥ 70% (Supplementary Material 1). An initial PPI network was constructed for these 156 orthologous protein sequences that resulted in the formation of a putative network with 95 nodes and 185 edges. Further, as a result of Agilent literature search (63 proteins) an additional 49 nodes with 34 edges were merged to the initial network (Supplementary Material 1) to get an extended parthenocarpy associated PPI network of 140 nodes (Proteins) with 219 edges (interactions) and designated as undirected network (Supplementary Fig. S1). Structural properties of the constructed network such as number of nodes, degree distribution, clustering coefficient were calculated (Supplementary Table S1) for better understanding the functional organization of proteins in the network. For example, the average connected component in the PPI network was found to be 22, indicating that the majority of the proteins in the network are highly connected that play a central role in the network’s architecture and considered to be essential proteins^34^. Further the degree distribution in this network approximates the power law (P (k) ~ (k – γ)) (P (k) ~ (59.5–1.3)) i.e., with smaller value of degree exponential (γ) − 1.3. This in turn determined the importance of hubs in the network i.e. networks with larger γ (< 3) value indicated that the hubs in the network are relevant to biological function rather behave like a random network. This meant that our network could perfectly reflect a biological network and thereby proteins in the network might efficiently communicate biological information related to parthenocarpy. In addition, the parthenocarpy-PPI network has a characteristics average path length value of 5 and comprised 40% shortest paths. This outlined the overall navigability of the network that the biological information in the network could get transferred by crossing few nodes from a selected protein to others in the network^35^. The clustering co-efficient of this scale free network is 0.283, that significantly describes that the internal structure of this network is highly interactive and form clusters.

### Topological analysis of parthenocarpy associated PPI network

Biological significance of proteins in this scale free network was determined by analyzing the centrality measures (topological properties) such as degree, betweenness and closeness centrality. Based on the topological properties of the constructed PPI network, top ten proteins with higher degree**,** higher betweenness centrality scores, higher closeness centrality score and higher clusters were taken and listed in Table 1. The average degree of proteins in the constructed PPI network was found to be 3.128 and proteins with high degree (> 10 interacting partners) such as *LFY, ZEP, HK2* (Histidine kinase CKI1)*, EXPA1* and *SL1* are referred as degree based hubs. Proteins with higher betweenness centrality scores such as *NIA1, ZEP, FL, NCED1, MOCOS* could act as useful indicators for detecting bottleneck protein in the PPI network. *LFY, FIE2, GAF1, NFYB9, ZEP* with high closeness centrality has a smaller path length to reach all other proteins in the network and thereby these proteins would have a greater influence in the network.

**Table 1:** Topological analysis—degree, betweenness, closeness centrality and cluster analysis of the network analyzed using Cytohubba plugin.

(A) Ranked by degree			(B) Ranked by betweenness method			(C) Ranked by closeness method			(D) Ranked by MCC method		
Rank	Node	Score	Rank	Node	Score	Rank	Node	Score	Rank	Node	Score
1	*LFY*	21	1	*NIA1*	3936	1	*LFY*	39.2159	1	*ZEP*	725,762
2	*ZEP*	12	2	*ZEP*	3694.22	2	*FIE2*	32.406	1	*GAF1*	725,762
2	*GAF1*	12	3	*LFY*	3602.76	3	*GAF1*	31.9071	3	*EXPA1*	725,761
4	*EXPA1*	11	4	*NCED1*	3534	4	*NFYB9*	31.0825	3	*RAP23*	725,761
4	*RAP3*	11	5	*MOCOS*	3480	5	*ZEP*	30.1262	5	*HK2*	725,760
6	*HK2*	10	6	*GAF1*	3319.09	6	*MADS2*	29.5825	5	*At4g13710*	725,760
6	*At4g13710*	10	7	*FIE2*	2807.91	7	*EMF2*	29.4159	5	*BAM1*	725,760
6	*BAM1*	10	8	*E2FB*	1994.8	7	*MAD16*	29.4159	5	*SL1*	725,760
6	*SL1*	10	9	*PHSH*	1609.17	9	*EXPA1*	29.1833	5	*GH3.8*	725,760
6	*GH3.8*	10	10	*ANT*	734.21	10	*AP2*	28.5762	10	*SCL7*	362,880

### Cluster analysis

Highly interconnected regions or sub network in parthenocarpy associated PPI were identified using MCODE plug-in since clusters in a network are often protein complexes which involved in the same pathway and the same protein family. Totally eight clusters were obtained and subjected to functional enrichment analysis using ShinyGO (Supplementary Fig. S2). Based on the biological process, Cluster 1comprises of genes belonging to the “Response to stress & Transcriptional regulation”, Cluster 2 belongs to “Histone modification” apart fromDNA repair, genes in Cluster 3 involved in Carbohydrate metabolic process, Oxido-reduction coenzyme metabolic process, Cluster 4, 6, 7 and 8 encompasses genes belonging to embryo, seed sac development, mitotic cell cycle, Gametophyte development, reproductive processes and hormonal regulation etc. Thus genes belongs to hormone-mediated pathway, Response to hormonal/chemical stimulus, Regulation of multicellular organism development and Regulation of gene expression” etc., are get highlighted as a result of MCODE analysis. Association of genes involved in parthenocarpy with stress mechanism was well reported in our previous review^36^ and thus the current study mainly focused in understanding the association of genes with respect to various hormonal signaling. The unique rankings of genes based on each centrality measures and MCODE clusters analysis are given in Table 1 and the genes belongs to each clusters as a result of MCODE plugin are given in (Supplementary Table S2).

### Functional enrichment and KEGG pathway analysis

Functional enrichment of the proteins in the overall network revealed that majority of the genes are primarily involved in floral whorl development (26.32%), meristem maintenance (15.79%), regulation of reproductive process (10.53%), transcriptional regulation, gene regulation (10.52%) and oligosaccharide biosynthetic process (10.53%). This in turn supported the relevance of orthologous genes short listed for the construction of PPI network in the current study since many studies reported the significance of genes involved in floral development, ovule integument, reproductive process etc., in seedless fruit formation^37,38^. Considering the functional and GO analysis of the constructed PPI network together, it was shown that majority of the genes that framed the PPI network are involved in “regulation of cellular macromolecule biosynthetic process” and “transcriptional regulatory activity” (Supplementary Fig. S3). Particularly MADS family transcription factors that are widely involved in the flower-fruit transition stage like *AGL8, MADS16, LFY*and *MADS29* were repeatedly found in all the three centrality measures as well as in cluster analysis and thus ranked as key genes. This is in correlation to our previous review that highlighted that MADS box transcription factors in parthenocarpic accessions could act as a key regulator in fruit set that mediate seedless fruit formation^36^. Next to the MADS box transcription factors, proteins involved in hormone regulatory mechanism (Histidine kinase (*HK2*), indole-3-acetic acid (IAA)-amidosynthetase GH3.8, DELLA (*RGA, RGL1, RGL2*), Gibberellin receptor (*GID1C*), Expansin (*EXPA1*) and cellular metabolism (ATP citrate synthase (*ACSB2*), zeaxanthin epoxidase (*ZEP*), leucine-rich repeat receptor-like serine/threonine-protein kinase (*BAM1*) scored next top ranks.

**Figure 2:**
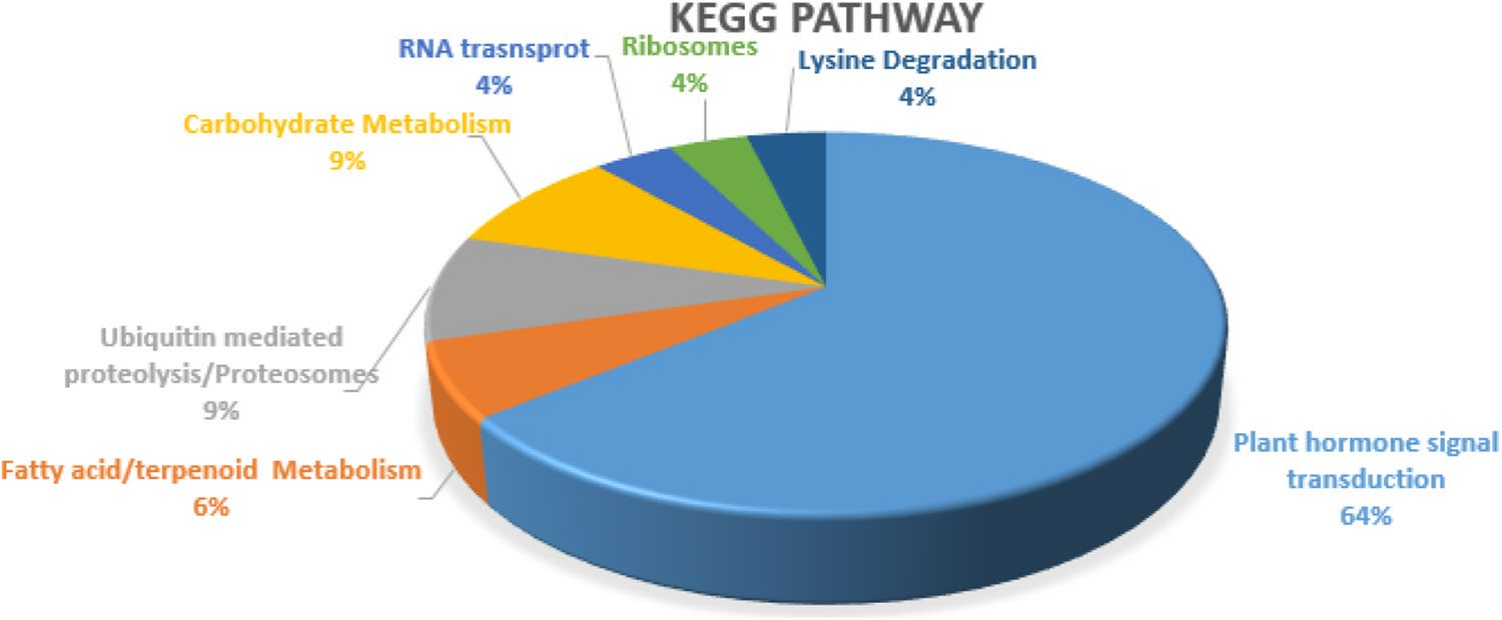
KEGG pathway analysis of parthenocarpy-PPI Network. Majority of the proteins in the parthenocarpy-PPI network are involved in plant hormone signal transduction (64%) followed by carbohydrate and ubiquitin mediated proteolysis (9%). Details of genes involved in the respective KEGG pathway are given in Supplementary Table S3.

Similarly KEGG pathway analysis rationalized that hormone signal transduction, carotenoid biosynthesis, fatty acid metabolism, carbohydrate metabolism and lysine degradation pathways having a strong association in the network of natural parthenocarpy (Fig. 2). In our previous review, the role of hormone mediated transcriptional regulation in parthenocarpy was emphasized, while the current study highlighted involvement of proteins in carbohydrate, fatty acid and lysine degradation pathways (Supplementary Table S3). It has been speculated that some of the discrete nature of parthenocarpic fruits such as its nutritional value, pulp content, fruit size, peel thinness etc., might be due to the cellular metabolism that occurs in parthenocarpic fruit formation. While considering the role of lysine degradation pathway, it was found that glycine and carnitine are the end products which incite us to acquire information regarding free amino acid (FAA) content difference in parthenocarpy and seeded varieties of *Musa* spp. Variation in the level of FAA between parthenocarpy and seeded traits were observed in tomato^39^. On the other hand, group of polycomb (PcG) proteins *MEA, FIE, CLF* and *SWN* are also highlighted under lysine degradation pathway.

### Validation of candidate genes for parthenocarpy

**Figure 3:**
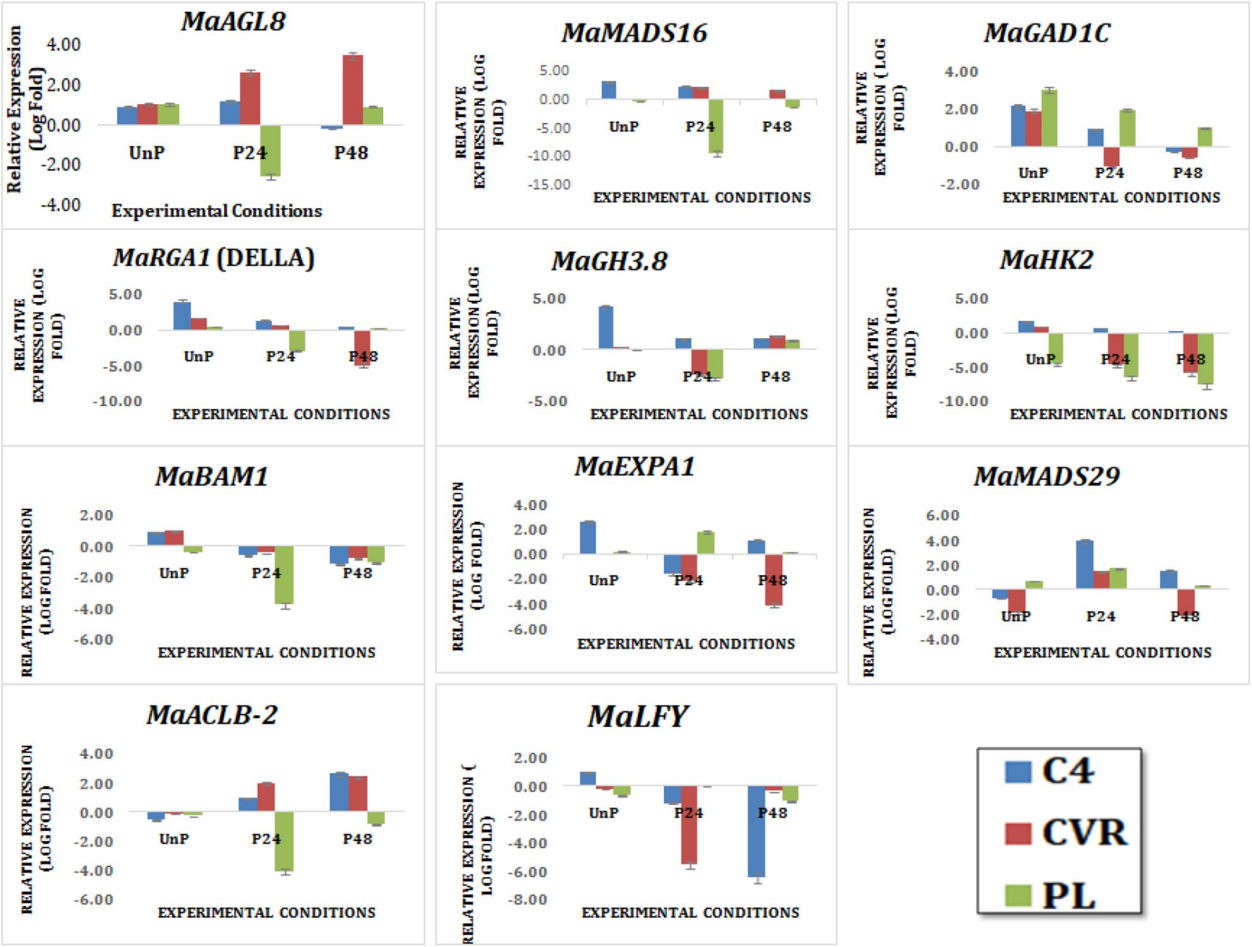
Relative expression of genes in ovary of three banana cultivars (C4, CVR and PL) at three conditions namely Un pollinated (UnP), 24 h after pollination (P24) and 48 h after pollination (P48). Relative expressions of genes with respect to experimental condition (X-axis) were expressed in Log fold change (Y axis). The mean differences between relative gene expressions were analyzed by ANOVA, p < 0.05. p values of the two-way ANOVA are shown in (Supplementary Material 2).

The shortlisted genes namely *ZEP, LFY, MADS29, GID1C, RGA1, HK2, MADS16, BAM1, GH3.8, AGL8, EXPA1,* and *ACLB2* were subjected to experimental validation using qRT-PCR (Table 2). Ovary samples of *Musa acuminate* ssp. *Burmaniccoides* (Calcutta 4 (C4)-profuse seed set), (cv. Rose (CVR)-set seeds upon pollination) and (Pisang Lilin (PL)-seldom setting seed) were collected at three different conditions namely un-pollinated (UnP), 24 h (P24) and 48 h (P48) after pollination for the present study. Expression analysis of the candidate genes using qRT-PCR is shown in Fig. 3. In UnP condition, *MaMADS16*, *MaGH3.8*, *MaLFY*, *MaEXPA1* and *MaRGA1* exhibited larger expression profile pattern in C4 whereas the expression pattern of these genes were similar in CVR and PL. Interestinglysimilar expression pattern of *MaBAM1, MaHK2* and *MaMADS29* were observed in both UnP ovaries of C4 and CVR. Contrarily, expression pattern of *MaAGL8, MaGID1C and MaACLB2* were alike in the three accessions under un-pollinated condition. Upon P24, expression level of the two MADS box transcription factors namely *MaAGL8* and *MaMADS16* were up regulated in C4 and CVR and down regulated in seedless PL. Though *MaMADS29* had shown increased expression in all the three accessions of C4, CVR and PL at P24, higher expression is observed in the seeded accession C4. This confirmed the negative regulation of MADS box transcription factors upon pollination in natural parthenocarpic accessions like PL. Negative regulation of *DELLA* in seedless fruit formation is well documented during artificially induced seedless fruit formation in various crops^40–47^. In the current study also threefold down-regulated expression of *MaRGA1 (DELLA)* was observed in PL compared to C4 and CVR at P24. Further it is interesting to note that, upon pollination, there was reduction in the expression level of *MaGID1C,* where the reduction was drastic in CVR followed by C4 and PL^48^. It is well known that external application of GA competes *DELLA* for its interaction with *GID1C* leading to subsequent degradation of *DELLA* eventually resulting in seedless fruit formation^50^. Reduced expression of *MaGID1C* and *MaRGA1* in pollinated ovaries of CVR confirmed their function in seed development. *IAA (GH3.2)* is an auxin-amino acid conjugating enzyme that converts auxin into an inactivate form, reported that homolog of *GH3.2*, (i.e. IAA-amino synthetase (*GH3.8*)) down regulates auxin signaling by preventing the accumulation of free *IAA*^50,51^. Downregulated expression of *AUX/IAA* and homologs of *GH3.2* was reported in parthenocarpic eggplant over the seeded eggplant^52^. Similarly down regulation of *MaGH3.8* was observed in the ovaries of both CVR and PL at P24. Expression study of histidine kinase (*MaHK2*) and *MaBAM1* shown that in PL and CVR, they were down regulated and the level of expression was very much lower in PL compared to CVR. Similar expression profile of these two genes in other seedless fruits such as tomato, eggplant and capsicum further evidenced its role in parthenocarpy^53^. GA induced parthenocarpy showed increased expression of expansin in the ovaries of pear fruit suggesting that genes involved in cell expansion, cell division get activated upon hormonal signaling for fruit^26^. Similarly *MaEXPA1* is up regulated in PL whereas it is down regulated in both C4 and CVR at P24. Increased expression of *MaACSB2* was evidenced in artificially induced seedless tomato^54^, however *MaACSB2* was down regulated in PL and up regulated in C4 and CVR upon pollination at P24. *MaLFY* (MADs TFs) another candidate gene reported to involve in floral meristem initiation^55^ was observed to down regulated in all the three cultivars upon pollination (both P24 and P48). This inferred that *MaLFY* might play a role in floral initiation rather than fruit and seed set. In addition, Ct value of the gene *MaZEP* was undetermined due to it’s in all three accessions irrespective of the conditions. These results from banana interrogated the significance of *MaLFY, MaACSB2* and *MaZEP* in natural parthenocarpy. Predominantly, genes such as *MaAGL8, MaGID1C, MaMADS16, MaMADS29, MaBAM1, MaHK2, MaGH3.8*, *MaRGA1*and *MaEXPA1* had similar expression pattern in *Musa* spp. as reported in other artificially induced parthenocarpic horticultural crops such as tomato^56^, eggplant^57^, pear ^58^ and apple^27^.

**Table 2:** Shortlisted candidate genes from the constructed PPI network for the trait parthenocarpy.

Accession ID (Version I)	Gene Name	Gene description retrieved using BLAST2GO^59^	Expression of gene in other seedless traits**	Expression in *Musa* spp.								
				C4			CVR			PL		
				UnP	P24	P48	UnP	P24	P48	UnP	P24	P48
GSMUA_Achr7P18880_001	*MaZEP*	Zeaxanthin epoxidase	Down regulation	*	*	*	*	*	*	*	*	*
GSMUA_Achr6P16390_001	*MaLFY*	LFY-like protein OrcLFY	Down regulation	++	--	----	-	----	--	--	-	--
GSMUA_Achr3P23580_001	*MaMADS29*	MADS-box protein AeAP3-2 isoform X1	Down regulation	--	++ ++	++	--	++ +	---	++	+++	+
GSMUA_Achr3P22920_001	*MaHK2*	probable histidine kinase 2	Down regulation	++	+	+	+	---	---	---	----	----
GSMUA_Achr9P20950_001	*MaMADS16*	MADS-box transcription factor 16-like	Down regulation	++	++	--	--	++	++	-	----	--
GSMUA_Achr4P07370_001	*MaBAM1*	leucine-rich repeat receptor-like serine/threonine-protein kinase BAM1	Down regulation	++	--	--	++	–	--	--	----	---
GSMUA_Achr4P07220_001	*MaGH3.8*	probable indole-3-acetic acid-amidosynthetase GH3.8	Down regulation	++++	++	++	+	---	++	+	---	++
GSMUA_Achr11P05030_001	*MaACLB-2*	ATP Citrate synthase beta chain	Up regulation	–	++	++ ++	-	++ +	+++	-	----	--
GSMUA_Achr3P02280_001	*MaAGL8*	Agamous like MADS TF	Down regulation	++	++	–	++	++ +	++++	++	----	++
GSMUA_Achr1P21300_001	*MaRGA1*	DELLA	Down regulation	+++	++	+	++	+	----	+	---	+
GSMUA_Achr8P05910_001	*MaGID1C*	Gibberellin 1C receptor like	Up regulation	+++	++	-	++ +	–	-	++++	+++	++
GSMUA_Achr1P02650_001	*MaEXPA1*	expansin-A11-like	Up regulation	+++	--	++	+	---	----	+	+++	+

**References included in Supplementary files.

* qRT-PCR expression values is “Undetermined”.

+  → Expression; ++ , +++ & ++++  → 2, 3 and fourfold expression.

- → Down regulation; --, --- & ---- → 2, 3 and fourfold down regulation.

## Discussion

**Figure 4:**
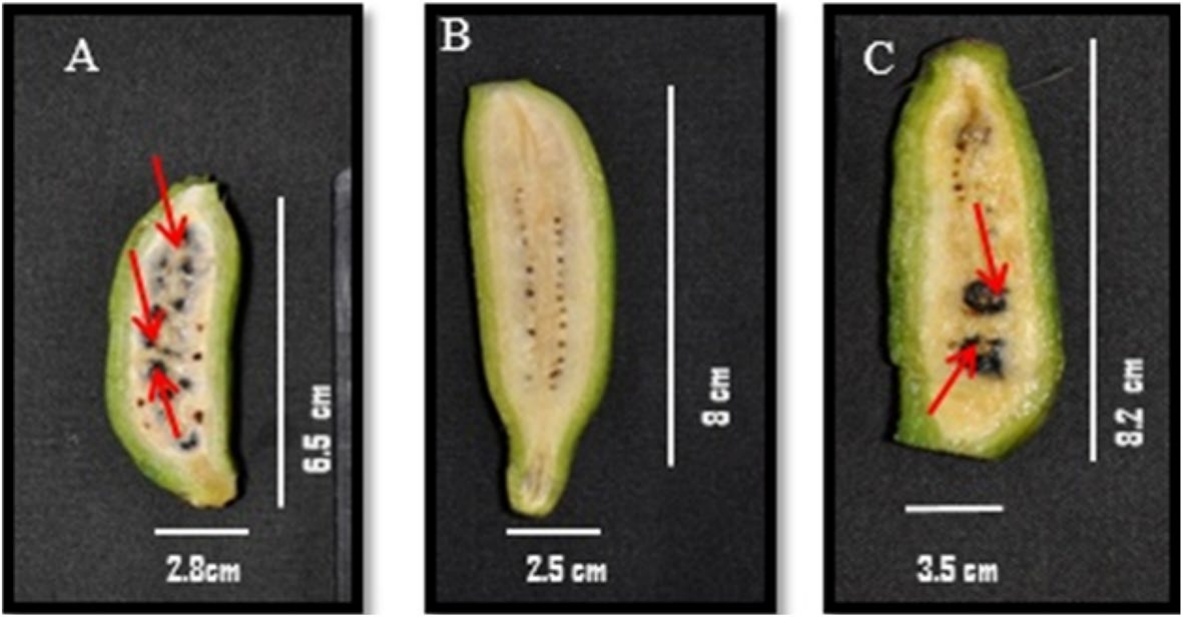
Fruit image of *Musa* spp. (**A**) C4 (IC no. 0642) profuse seed set, (**B**) PL (IC no. 0195) seldom setting seeds upon pollination, (**C**) CVR (IC no. 0638) parthenocarpic accession, upon pollination rarely setting seeds occurs respectively. Arrows indicate location of seeds in seeded accessions (*Musa* spp.).

Till date candidate genes reported for parthenocarpy in different crops are only based on artificially induced parthenocarpy but not on natural parthenocarpy as in banana. Ergo, in the current study we made an attempt to identify the candidate genes for understanding the molecular mechanism of natural parthenocarpyin *Musa* spp. Based on the fact that orthologous sequences are ought to have the same functions^60^, the computational method of PPI network based mining was employed. This leads to the findings of *ZEP, LFY, MADS29, GID1C, RGA1, HK2, MADS16, BAM1, GH3.8, AGL8, EXPA1,* and *ACLB2* as candidate genes for parthenocarpy. Consequently to confirm role of these candidate genes in *Musa* spp. we performed two approaches: in vitro expression analysis followed by exploring their interactions with other proteins through in silico. In the first approach, factors that loomed large the seed set like genome groups, partial sterility, pollination and period of fertilization etc., were taken into consideration for cultivar selection in the current study.It is reported that parthenocarpy in banana is governed by “A” genome^28,30^, while the dynamic nature of AA genomic accessions exhibiting both seeded (C4**—**Fig. 4A) and seedless traits (PL—Fig. 4B)was also been reported^61^. Among the seedless AA diploid accessions, some are amenable (cv. Matti, cv. Rose (Fig. 4C)) and few are recalcitrant (PL) to seed set upon artificial pollination^62^. In view of this, validation of candidate genes through qRT-PCR was carried out in three diploid ‘AA’ accessions (C4-with profuse seed set; CVR and PL-setseeds moderately and rarelyrespectively upon pollination) at three different time intervals-UnP, P24 and P48. The results of expression studies inferred that (i) Majority of the variations were observed between 24 h after pollination (P24) and UnP in CVR and PL but not in P48. This is in line to our earlier findings that male gametes reach the ovule within 24 h after artificial pollination^63^ and so we suggested that ovary sampling around 24 h after pollination is optimum for seed set studies in AA genome accessions of *Musa* spp. (ii) Similar expression pattern of *MaAGL8, MaMADS16, MaRGA1, MaBAM1* and *MaEXPA1* in P24 samples of CVR and C4 whereas except *MaBAM1*, similar pattern of expression were observed in UnP of CVR and PL. In a nutshell, the observance from this expression study inferred that CVR behaves more like the seeded accession C4 under pollinated condition. (iii) Down regulation of genes related to residual fertility (*MaHK2, MaGH3.8 and MaMADS29*) in P24 and P48 of CVR and PL further inquired residual fertility and fewer number of seed set in CVR compared to C4. In contrast to this, two fold down regulation of *MaGID1C* in P24 of CVR alone speculated their importance in seed set. (iv) As reported in seedless fruit formation of other crops, reduced expression of *MaAGL8, MaMADS16, MaRGA1, MaBAM1* and higher expression of *MaGID1C, MaEXPA1* were observed in P24 of PL compared to C4 and CVR drawn attention as key genes for seedless fruit formation in *Musa* spp.

**Figure 5:**
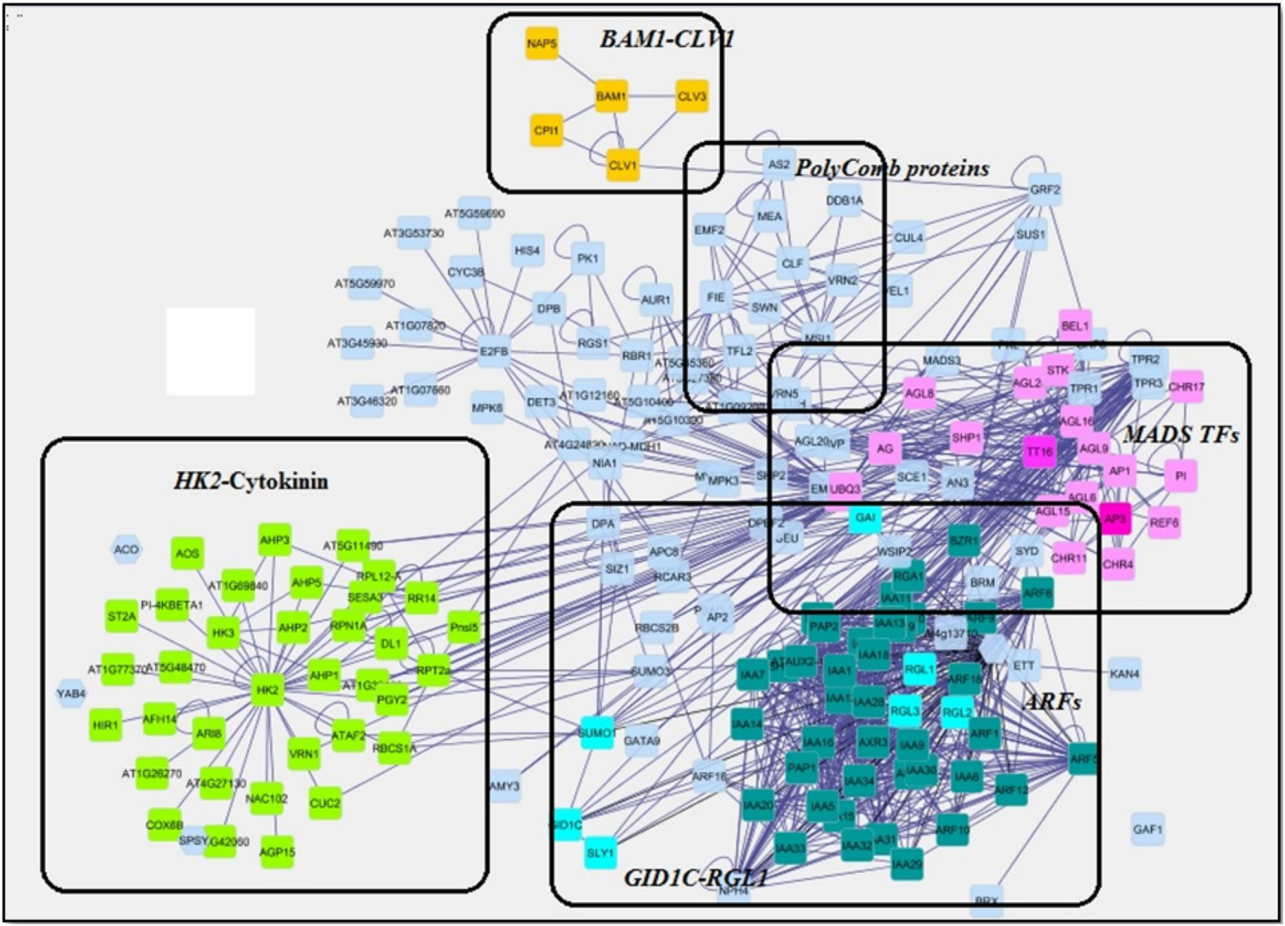
Association of validated candidate genes in the constructed PPI network.

**Figure 6:**
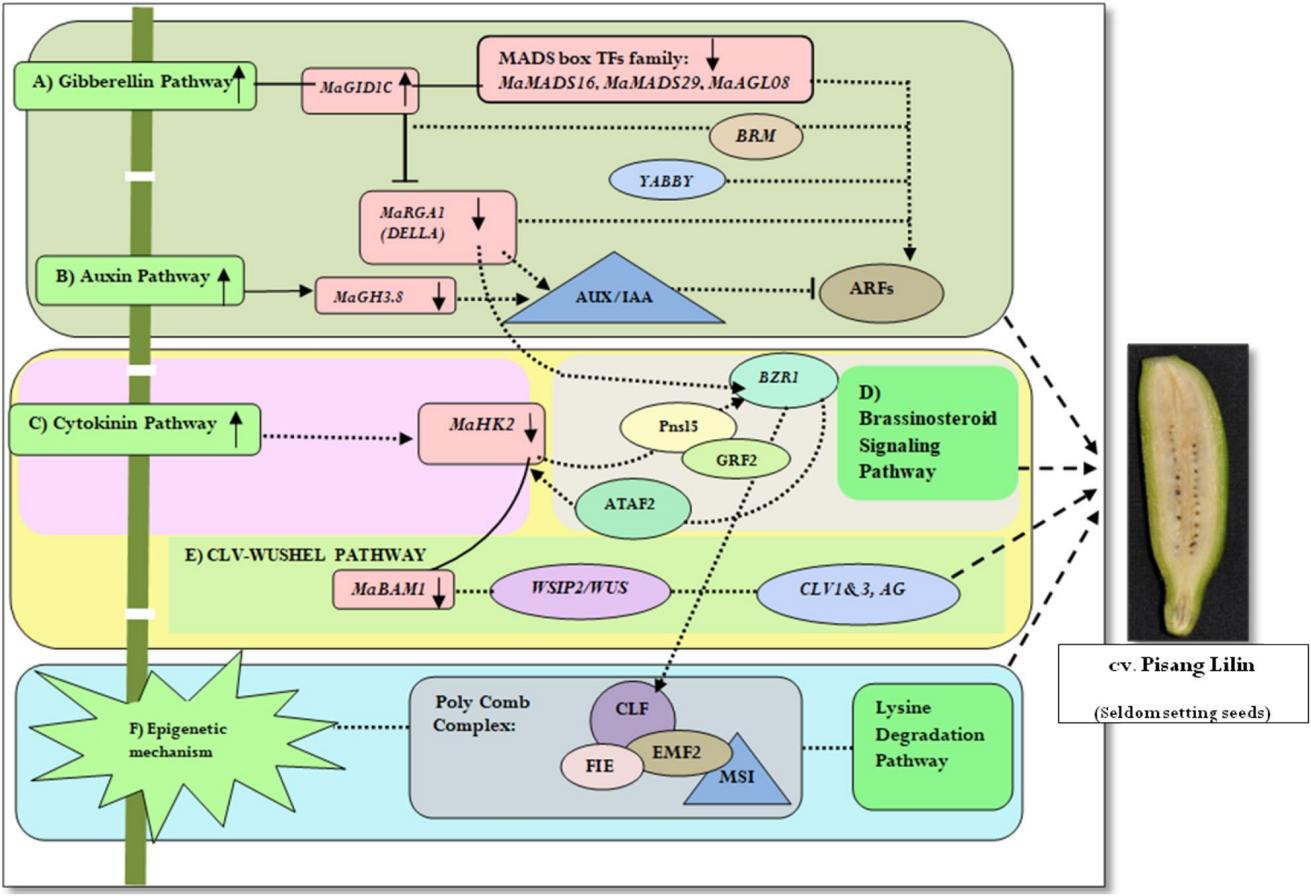
Putative pathway for natural parthenocarpy in *Musa* spp. (**A–C**) Increase in auxin, GA and Cytokinin induces parthenocarpic fruit formation by down regulating the expression of MADS box TFs (*MaAGL8, MaMADS16, MaMADS29*), *MaRGA1 (DELLA), MaHK2* and *MaBAM1*. MADS box TFs, *MaRGA1* and *BZR1*c ould act as a focal point in auxin mediated GA response in parthenocarpy. (**D**) Genes related to brassinosteroid signaling pathway (*BZR1*) get interacts with *Pnsl5* and *GRF2*act asa bridge between *MaHK2, MaBAM1* and *CLV-WUS* signaling pathway that induces seedless fruit formation. (**E**) CLV-WUSHEL signaling pathway where *WSIP2* is the wushel interacting protein get interacts with *WUS*, *CLV1*, *AG* and thereby associated with *MaBAM1* which issignificantly down regulated in parthenocarpic accession cv. Pisang Lilin*.* (**F**) Group of poly comb proteins (*CLF, FIE, EMF2, MSI—*histone modifying enzymes) which arereported to be involved in epigenetic mechanism of reproductive development. Their association to *MaBAM1* (involved in male/female gametophyte development) and *CLV* speculated their role in seedless fruit formation. KEGG analysis also highlighted that these genes are involved in lysine degradation pathway. Green color box indicates hormonal signaling pathway and epigenetic mechanism that are predicted to mediate seedless fruit formation; Dotted arrow lines represents the putative flow and the association of interaction partners to the validated genes which is derived from the constructed PPI network; Highlighted genes in rounded rectangle boxes along with ↓ symbol indicating down regulated expression pattern in cv. Pisang linin upon pollination (P24) in the current study. Highlighted gene in rounded rectangle boxes along with ↑Symbol indicated increased expression pattern *MaGID1C* in cv. Pisang Lilin. Genes mentioned in the triangle boxes are literature derived genes where corresponding supporting evidences were given in the discussion section.Genes mentioned in the oval boxes are retrieved based on their interaction with candidate genes in the constructed PPI network; Where expression of these genes *MaAGL8, MaMADS16, MaMADS29*, *MaRGA1 (DELLA), MaHK2, MaBAM1, MaGID1C, MaGH3.8* (mentioned in the pathway) were validated in three cultivars C4, CVR and PL of *Musa* spp.

Among the predicted12 candidate genes, nine genes *MaAGL8, MaMADS16, MaGID1C, MaRGA1, MaGH3.8, MaHK2, MaBAM1, MaEXPA1* and *MaMADS29* could be taken as candidate genes for the further study of natural parthenocarpy in *Musa* spp. By exploring the protein–protein interaction of the validated 9 candidate genes and their associations in the constructed PPI network (Fig. 5), we proposed a hormone mediated putative model that brings insight the underlying mechanism of parthenocarpy in *Musa* spp. (Fig. 6).

Distinct regulation of active hormonal signaling of gibberellin and auxin followed by the down regulation of MADS box TFs were reported so far^64^, however the functional associations of these genes and hormonal signaling in seedless fruit formation is still unclear. The constructed PPI network provides a way to visualize interrelation among the MADS box TFs and their functional association with auxin, GA and brassinosteroid hormone signaling. Probing the association of MADS box TFs in the network revealed it’s interaction with auxin responsive genes like *IAA11, ARF18* in which the association of MADS TFs with *BRM *(transcription regulatory protein *SNF2*) and *BZR1 *(Brassinosteroid signaling positive regulator protein family) in the network act as a bridge between MADS box TFs and auxin responsive genes^65^ (Supplementary Fig. S4A). Further interaction of *RGA1* (DELLA) to *GID1C; BZR1; ARF6* in the network prompted the crosstalk between gibberellin, auxin and brassinosteroid signaling (Fig. 6A,B).

Expression pattern of candidate genes like *MaGID1C, MaRGA1*(DELLA) *and MaGH3.8* in *Musa* spp. and their association in the constructed PPI network strongly supported our earlier review stated that increased GA together with decreased expression of auxin responsive genes like *GH3.2* resulting in seedless fruit formation^54,66^^.^ It is also evidenced that study on *DELLA* and its interaction with *ARF7/IAA9*, shown that *GH3.2* is the direct targets of *DELLA/ARF7-IAA9* which involved in the regulation of auxin homeostasis through GA during fruit development^17^*.* Further association of MADS TFs with auxin responsive genes is confirmed by a study related to silencing *PIN* (an auxin efflux transport protein) in tomato^42^. Upon critical evaluation of *BRM, BZR1, GH3.8, GID1C and DELLA* for its association with MADS box TFs and ARFs in the PPI network, we proposed direct or mediated interactions of MADS box TFs, and ARFs with *DELLA*, *BRM, GH3.8, GID1C and BZR1* speculating GA mediated auxin signaling in seedless fruit formation (Fig. 6A,B). Besides GAs and auxin, cytokinin and brassinosteroids (BRs) also plays a critical role in parthenocarpic fruit development, however the hormonal crosstalk associated with these hormones remains mysterious. Significant down regulation of *MaBAM1* particularly in seedless accession (PL) made us to look into theinteraction partners of *BAM1*-*CLV3, CLV1, CPI1* and their connectedness to other hubs in the network. To our surprise, interaction of *CLV1* with *Pnsl5, BZR1, WSIP2* (WUS-interacting protein 2) and *GRF2* explored its association with *HK2, MADS TFs and ARFs* (Supplementary Fig. S4B). Though association of *HK2* with *BAM1* is not elusive, an earlier study based on comparative genomic approaches in *Musa* spp.reported the role of *HK2* in gametophyte development^67^ and another study in *Arabidopsis* reported that *BAM1/2* as important regulators of anther development^68,69^. Further when queried the association of *BAM1*, *CLV1* with *WSIP2* (WUS—interacting protein 2) and MADS TFs, there are several reports that highlighted the role of cytokinin mediated *WUS* signaling^69–73^ and its association with clavata, *AG*^74–77^ (Agamous like MADS TFs) in ovule development. In addition, down regulation of *BAM1*/*/2* and WUSCHEL (*WUS*) in the mutant seedless tomato that exhibit both male and female sterility^68^ and the role of *WUSCHEL* in mediating the expression of *CLV3* and *AG* during floral development particularly in the ovule and integument formation^78,79^ received significant attention. From these findings we proposed a model for cytokinin mediated CLV–WUS signaling pathway in parthenocarpic fruit set through regulating male and female sterility in association with *BAM1, HK2* and MADS TFs (Fig. 6C–E). On the other hand, further experimental validation needs to carry out in order to clarify the functional association of CLV–WUS signaling in seedless banana fruit formation.

A group of proteins that are highlighted separately in the proposed pathway are polycomb (PcG) proteins namely *MEA, FIE, CLF and SWN* (Fig. 6F). KEGG pathway analysis of these proteins in the network showed their association with lysine (a free aminoacid) degradation pathway. Since interaction of MADS box TFs namely *AP2, AGL15, AGL2/EMF*, with a cluster ofPcG proteins namely *MSI1, FIE, SWN* and *VEL1* through *VRN5* (Vernalization 5) and *TPL* (transducing family protein/WD-40 repeat family protein) drawn attention in the network. Down regulation of *VRN5, TPL* as well MADS TFs and PcG proteins were already reported in parthenocarpic fruit development^65^ suggesting their promising role (Supplementary Fig. S4C).While considering the role of lysine degradation pathway, it was found that glycine and carnitine are the end products which incite us to acquire information regarding free amino acid (FAA) content difference in parthenocarpy and seeded varieties of *Musa* spp. Variation in the level of FAA between parthenocarpy and seeded traits were analyzed in tomato and capsicum but still the possible role of FAA content in parthenocarpic fruit formation is yet to prove. Despite these reports, the direct role of polycomb (PcG) proteins in lysine degradation pathway remains unclear in seed development and understanding the integration of these genes and the pathways in parthenocarpy is the key challenge. Besides, PcG proteins are act as histone modifying enzymes and reported to regulate the embryo and endosperm proliferation and anterio-posterior organization during seed development^80^. The possible role of epigenetic mechanism of these polycomb proteins in plant reproductive development particularly from flower to seed development is well reported earlier^81^. Another unpublished work at ICAR-NRCB, reported failure of certain female fertile accessions to set seeds under a set of environmental condition but the reason behind this behavior remains undiscovered. Thus it is speculated that PcG might be involved in epigenetic mechanism that regulates the seed formation under specific environmental conditions.

In a nutshell, the findings in the current study brings insight into hormone mediated pathway in seedless fruit formation as well as arouse a thirddimension approach to study the role of epigenetic mechanism and the level of free amino acids in seeded and seedless accession of *Musa* spp. We also suggested *MaMADS16, MaAGL8, MaDELLA, MaGID1C, MaGH3.8, MaHK2, MaBAM1, MaMADS29* and *CLV1* could be the possible target genes for manipulation of seeded accessions to parthenocarpy in *Musa* spp.

## Materials and methods

The approach used in this study for prioritizing key genes in parthenocarpy is summarized and described in the following sections.

### Mining of genes associated with the trait parthenocarpy

Genes associated with parthenocarpy in other crops were mined from databases like Uniprot, KEGG and sources like Pubmed, Pubmed Central, etc. This is achieved through manual text mining by using the query words “parthenocarpy’, “seedlessness”, “parthenocarpy and genes”, “parthenocarpy and transcription factors”, “parthenocarpy and *Musa*”. In addition, highly enriched differentially expressed genes (DEGs) between seeded and artificially induced parthenocarpic fruits either through phyto-hormone or chemical spray/mutation/genetic transformation in various crops such as tomato^59^, eggplant^33,52^, apple^27^ pear^27,58^ etc., were extracted from their respective transcriptome profiles (Supplementary Material 1).

### Retrieval of orthologous sequences in *Musa* spp.

The corresponding sequences pertaining to the mined parthenocarpic genes were downloaded in fasta format either from Uniprot (https://www.uniprot.org/) or from the respective crop specific genome or transcriptome databases using their unique reference gene ID cited in the literature. These sequences were then submitted to BLAST search in Banana Genome hub (http://banana-genome-hub.southgreen.fr/) ^82^ in order to retrieve corresponding orthologous sequences in *Musa* spp. which has ≥ 70% sequence identity (Supplementary Material 1).

### Construction of protein–protein interaction network (PPI)

The retrieved *Musa* orthologous sequences were submitted to STRING v10.5 (https://string-db.org/), a pre-computed database for the exploration of PPI^83^. Predicted protein association networks with a combined score of > 0.4 were taken for the construction of PPI network using Cytoscape 3.7.1^84^. Since the initial PPI network constructed using STRING database had limited number of nodes (proteins) and edges (interactions) for the study, we extended our search of possible interacting partners for the extracted genes using the plugin called Agilent Literature Search ^85^ in Cytoscape (Supplementary Material 1).

### Topological and cluster analysis of the network

The extended PPI network is considered as an undirected graph (G) constituting the components V and E, in which proteins are denoted as nodes (V) and the interactions are represented as edge (E). In the current study, to identify key proteins from the network, topological properties such as degree (k), betweenness centrality (BC) and closeness centrality (CC) were analyzed. These three different centrality measures were calculated using CytoHubba, a Cytoscape plugin^86^ that explored nodes with high degree, high BC and CC to identify the important proteins related to parthenocarpy from PPI network. Cluster analysis was performed using Molecular Complex Detection (MCODE)^87^ plug-in which provides a novel clustering algorithm to screen the modules of the PPI network for parthenocarpy (parthenocarpy-PPI) through Cytoscape^87^ MCODE scores of > 3 and the number of nodes > 3 were set as cutoff criteria with the default parameters (Degree cutoff ≥ 2, Node score cutoff ≥ 2, K-core ≥ 2 and Max depth = 100). Genes identified from the clusters and the top ten genes from topological analysis were subjected to Gene ontology (GO) and KEGG pathway^88^ enrichment analyses using BinGO^89^, ClueGO^90^ and BLAST2GO^91^ in order to expedite the functional annotation of each genes.Functional enrichment of cluster of genes as a result of MCODE plugin were carried out using ShinyGO v0.61^92^. Interacting partners of the candidate genes mined as a result of network topological analysis were explored using the plugin Bisogenet^93^ in Cytoscape in order to fathom their mechanism in parthenocarpy.

### Identification and collection of plant materials

The test samples were collected from the field Musa genebank of ICAR-National Research Centre for Banana (NRCB), Tiruchirapalli, Tamil Nadu, India where more than 300 Indian accessions and 121 exotic accessions are being maintained. The tissue cultured propagules (AA genomic group) of the seeded accession (C4) and parthenocarpic accessions (PL & CVR) were received from the International Transit Centre (ITC), Belgium through ICAR-National Bureau of Plant Genetic Resources (NBPGR). Exotic collections (EC) numbers were given to the exotic introductions by ICAR-NBPGR and the details for the three cultivars used in the current study are provided below in the Table 3.

**Table 3:** Accession number, genomic and parthenocarpic nature of the accessions used in the study.

Accession name	Accession number	ITC number	Genomic group	Nature of the group
Calcutta 4	0654	ITC 0249	AA	Seeded (profuse seed set)
PisangLilin	0195	ITC 1121	AA	Parthenocarpy (seldom setting seeds)
Cultivar Rose	0638	ITC 0712	AA	Parthenocarpy (rarely setting seeds upon pollination)
Matti	0182	–	AA	Parthenocarpy

**Figure 7:**
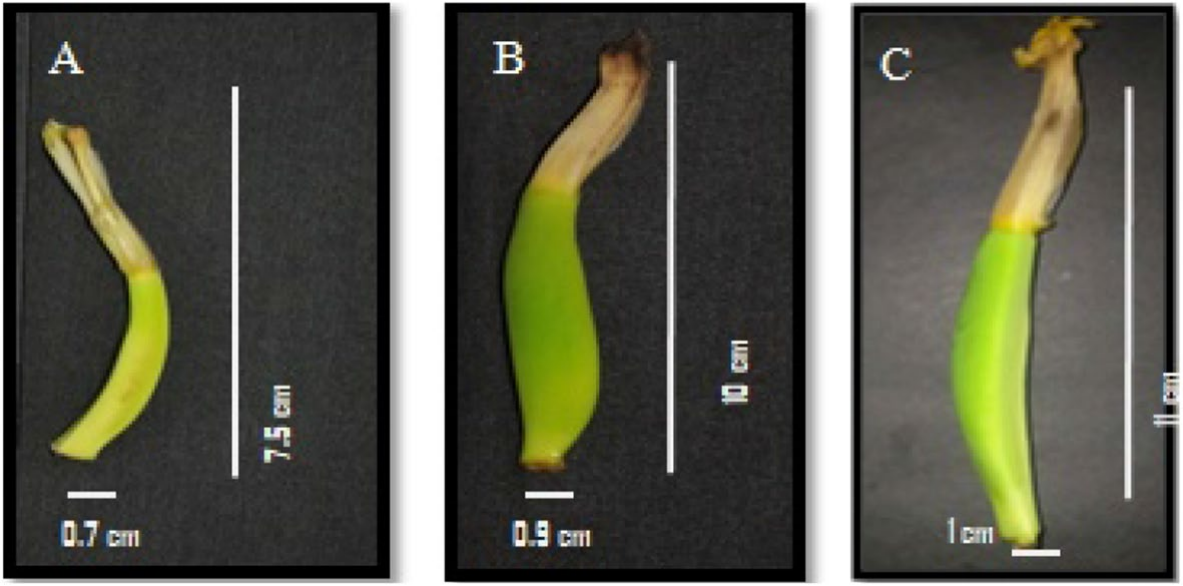
Ovary image of *Musa* spp. (**A**) C4 (IC no. 0642) seeded, (**B**) PL (IC no. 0195) parthenocarpic accession, very rare to set seed upon and (**C**) CVR (IC no. 0638) parthenocarpic accession, upon pollination seed set occurs respectively.

As a standard protocol, this has been deposited with ICAR-NBPGR for in-vitro maintenance. Tissue culture plants of these test accessions were sub cultured in the rooting media and the rooted plantlets were acclimatized through primary and secondary hardening under green house. The secondary hardened plants were planted in five replications with five plants per replication in the ICAR-NRCB field and maintainedat field conditions (Temperature 39 °C/27 °C and humidity 40%/85% day/night) and sufficiently watered for 60 days for plant acclimatization.At flowering time, the whole inflorescence was bagged before opening of the first female hand. The female floret of C4 (Fig. 7A), PL (Fig. 7B) and CVR (Fig. 7C) on the day of flower opening at 8.00am were collected and designated as un-pollinated (UnP) sample. For pollen grains (Male), Matti (AA) a local landrace collected from Thirunelveli, Tamil Nadu, India which is being maintained at ICAR-NRCB with accession number 0182 was taken. The pollen grains were collected during anthesis at 7.00 am from the accession Matti (Male), dusted over thestigma of the female florets (C4, CVR and PL) and the whole inflorescence was covered. The female florets collected at 24 h and 48 h after pollination were designated as P24 and P48 samples. ICAR-National Research Centre for Banana, Tiruchirapalli, Tamil Nadu, India being the National Active Germplasm Site (NAGS), all national and international guidelines and legislations were followed in performing the experimental research, field studies and collection of experimental samples.

### Ovary sample preparation

**Figure 8:**
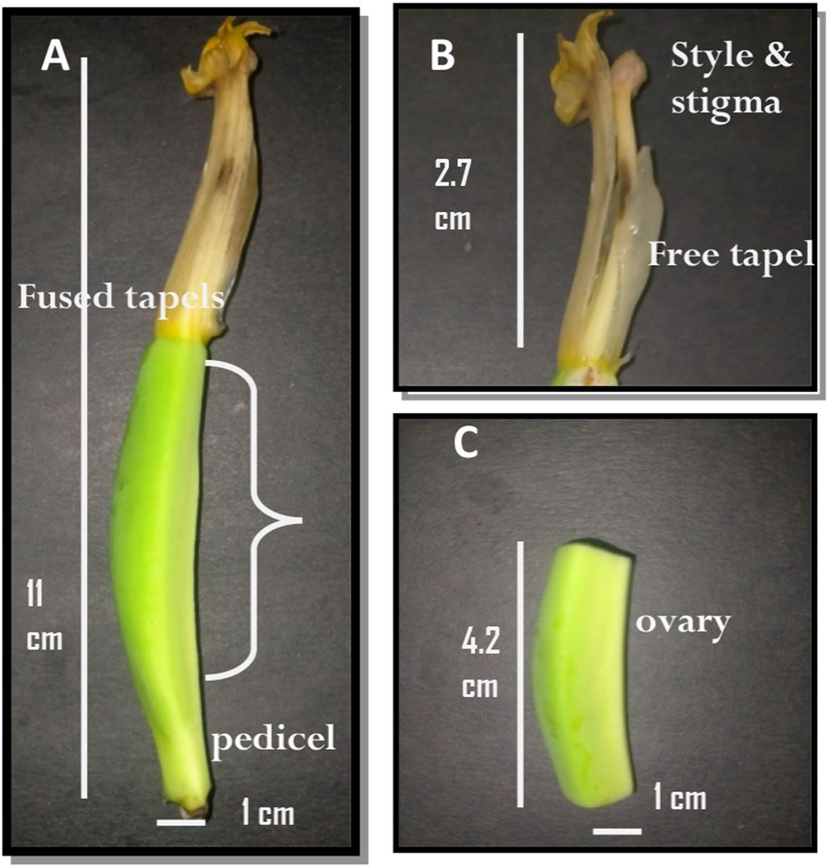
Image of banana female florets. (**A**) Full view of female florets, (**B**) style and stigma region of female florets, (**C**) ovary region of the female florets taken for RNA isolation.

The collected female floret (style, stigma, tepal and pedicel (Fig. 8A,B)) of C4, CVR and PL under pollinated and UnP conditions were cleaned with nuclease free water, immediately snap frozen and stored frozen − 80 °C. For total RNA isolation only the ovary part of the sample (approximately ¾th from the tip of the banana flower) as shown in Fig. 8C were taken**.** Initially, all the materials used for RNA isolation were treated with DEPC (diethyl pyrocarbonate) water.

### Validation of genes using qRT-PCR

From the result of topological and cluster analysis of parthenocarpic PPI network, we selected 12 candidate genes and subjected to qRT-PCR to compare their relative expression in seeded and parthenocarpic accessions of *Musa* spp. Total RNA of each sample was extracted using RNeasyPlantmini kit (Qiagen, Hilden, Germany) (product no. 74904) and the quantity /integrity of the RNA was checked using nanodrop (ColibriMicrovolume Spectrometer-Titertek/Berthold). The cDNAs of each RNA sample was synthesized using Transcriptor First strand cDNA synthesis kit (Roche) according to the manufacturer’s instructions. Primers for the experiments were designed using IDT-Primer design tool (https://www.idtdna.com/pages/tools/primerquest) and the primer sequences are provided (Supplementary Table S4). The qRT-PCR reactions were performed in triplicates with Ribosomal protein S2 (RPS2) as endogenous control and repeated thrice on three biological replicates and runon Light-Cycler 96 instrument (Roche Co. Germany) using the SYBR Green Master Mix (Thermo Fisher Scientific, USA). For each primer, 10 µl of reaction volume was set with 5 µM of both forward and reverse primer, 5 µl of 2× master mix and the final volume was made up with nuclease free double distilled water. The endogenous reference gene (RPS2) was used as an internal standard. Thermal cycling was performed as follows: 95 °C for 10 min (1 cycle); 95 °C for 10 s, 57–62 °C for 30 s, 72 °C for 20 s (45 cycles). At the end of PCR, the transcriptional expression level of each gene was quantified based on normalized ratio with advanced relative. The relative expression of each gene was determined based on comparative delta-delta CT method (ΔΔCT)^94^. Statistical significance analysis of expression values of candidate genes was performed using Data Analysis Toolkit in Excel based on one-way ANOVA (p < 0.05) (Supplementary Material 2)^39^.

## Supplementary Information

Supplementary Legends.

Supplementary Figures.

Supplementary Material 1.

Supplementary Material 2.

Supplementary Tables.
