# Supplementary material for: Decoding the molecular mechanism of parthenocarpy in Musa spp. through protein–protein interaction network
Source: Sci Rep. 2021 Jul 16;11:14592. doi: 10.1038/s41598-021-93661-3 (PMC8285514; doi:10.1038/s41598-021-93661-3)
Supplement: Supplementary file 1 — Supplementary Legends. [file 41598_2021_93661_MOESM1_ESM.docx]

**Electronic supplementary material**

**Supplementary Material 1. Worksheet 1:** List of Up-regulated; **Worksheet 2:** List of down-regulated genes retrieved through literature mining; **Worksheet 3:** List of genes retrieved through Agilent literature search plug in – Cytoscape app; **Worksheet 4:** BLAST Similarity result for orthologous genes in *Musa* spp. for the literature derived genes

**Supplementary Figures: S1**. Protein-protein interactions (PPI) of genes associated with Parthenocarpy (parthenocarpy-PPI); **S2**.Biological process of genes present in all the clusters obtained through MCODE plugin; **S3**. Functional characterization of genes in the parthenocarpy-PPI network identified via Cytoscape - ClueGO plug-in; **S4.** Possible interaction partners of validated genes in the network and their association in hormonal signaling.

**Supplementary tables: S1**. Structural properties of the constructed parthenocarpy-PPI network; S**2**. Details of 8 clusters obtained via MCODE plugin;**S3**. Details of KEGG pathways associated to parthenocarpy; **S4**. Details of the parthenocarpic candidate genes as a result of parthenocarpy-PPI network analysis and their primer sequences used for validation through qRT-PCR.

**Supplementary Material 2.** Statistical significance of relative expression of genes (ANOVA)
