# Supplementary material for: Decoding the molecular mechanism of parthenocarpy in Musa spp. through protein–protein interaction network
Source: Sci Rep. 2021 Jul 16;11:14592. doi: 10.1038/s41598-021-93661-3 (PMC8285514; doi:10.1038/s41598-021-93661-3)
Supplement: Supplementary file 2 — Supplementary Figures. [file 41598_2021_93661_MOESM2_ESM.docx]

***Supplementary Information for***

**Decoding the molecular mechanism of parthenocarpy in *Musa spp*. through protein-protein interaction network**

**Suthanthiram Backiyarani, Rajendran Sasikala, Simeon Sharmiladevi, Subbaraya Uma ***

**ICAR-National Research Centre for Banana, Thogamalai Road, Thayanur Post, Tiruchirapalli 620 102, Tamil Nadu**

*** Correspondence: UmaSubbaraya (**[**umabinit@yahoo.co.in**](mailto:umabinit@yahoo.co.in)**)**

**Telephone No: +91- 0431 2618125**

This Supplementary Information includes information on threefigures, four tables.

**Supplementary Figure S1**


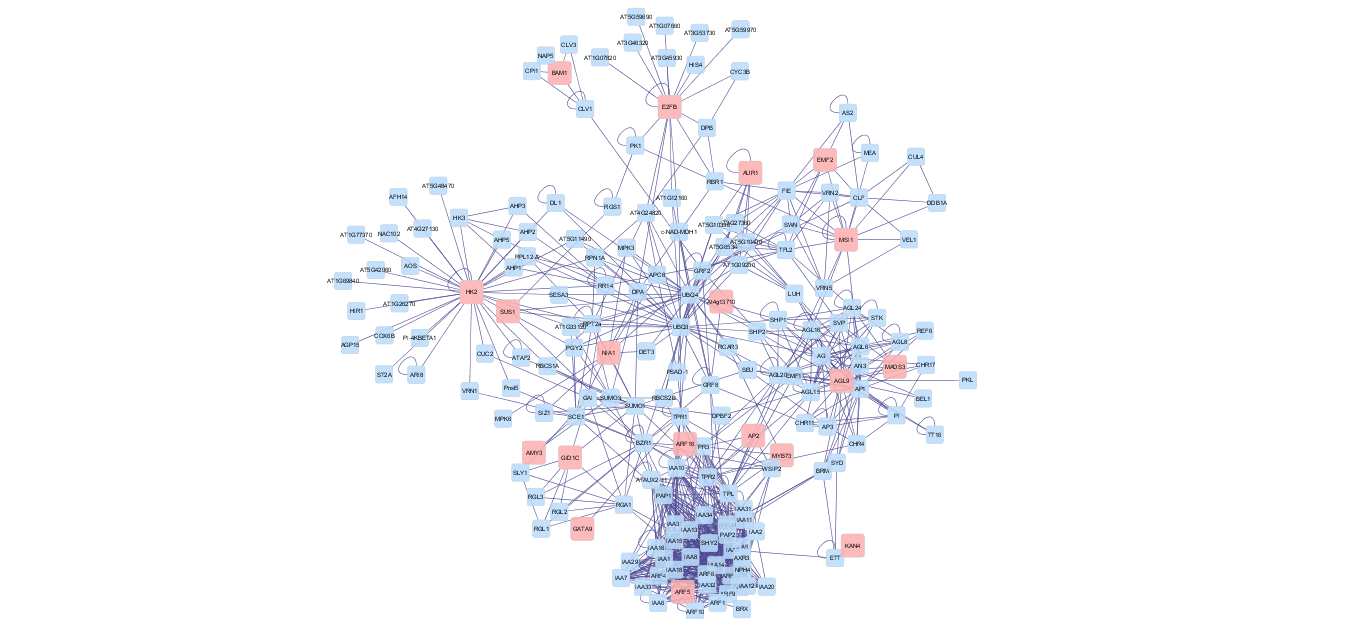


**Fig. S1: Protein-protein interactions (PPI) of genes associated with Parthenocarpy (PA-PPI) constructed using Cytoscape 3.7^76^and displayed using an edge-weighted spring embedded layout. Gray edges indicate protein interactions and square boxes indicate node (Gene/Protein). Genes that are shortlisted as top candidate genes are displayed in brown color where as the genes that interacts with candidate genes are in blue color.**

**Supplementary Figure S2**

**
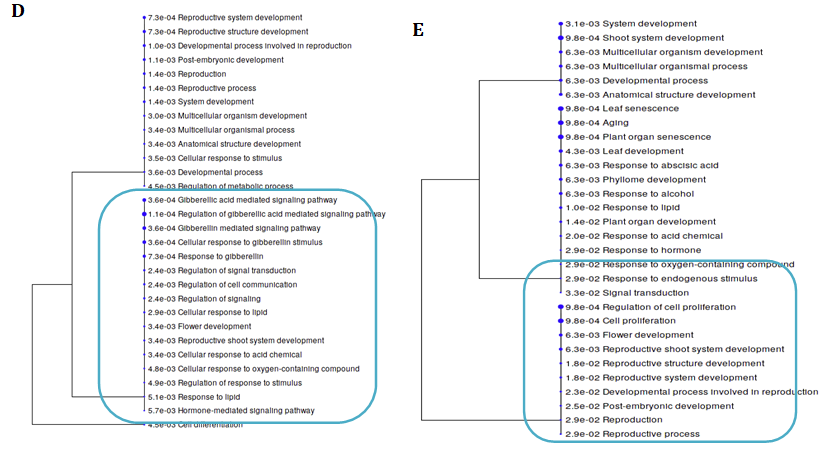

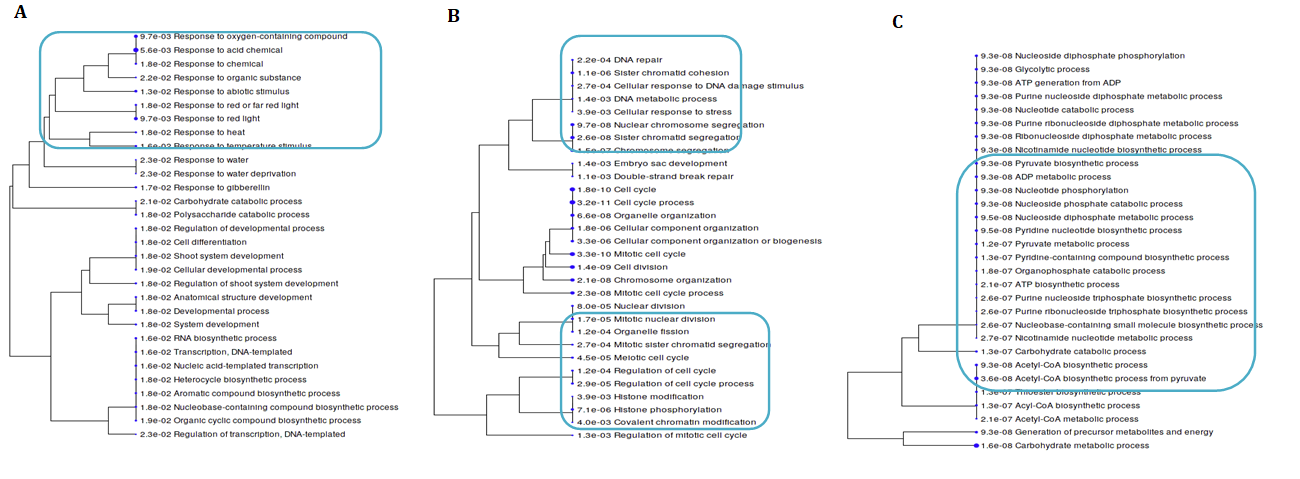
**

**
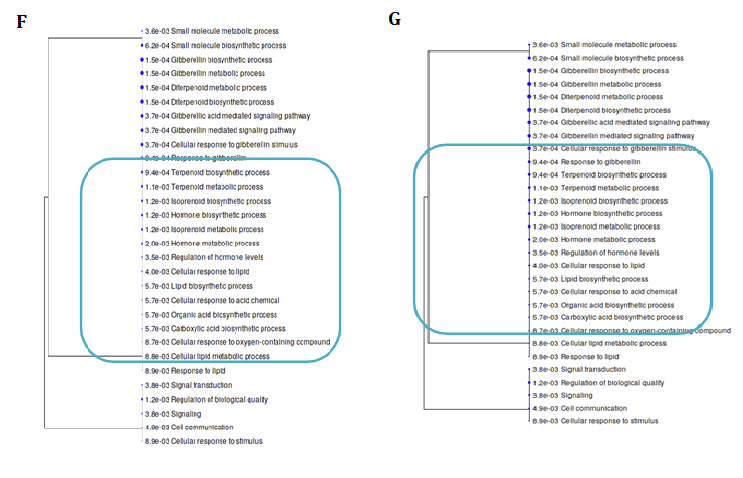
**

**Fig. S2: Biological process of genes present in the all the clusters as a result of MCODE^79^ plugin was performed using ShinyGO^84^. A) Genes involved in cluster -1 where genes related to stress responses are highlighted; B) Cluster-2- genes related to Histone modification and DNA repair mechanism; C) Cluster-3- genes related to Carbohydrate metabolic process are highlighted ; D) and E) cluster -4 and cluster 6 – where genes related to Reproductive development and signal transduction are highlighted; F) and G) cluster 7 and cluster 8 where genes related to hormonal signaling. Note: Biological process of cluster 5 – since only MADS TFs were involved (*MAD16, MAD2, AP2*), GO analysis couldn’t perform.**

**Supplementary Figure S3**


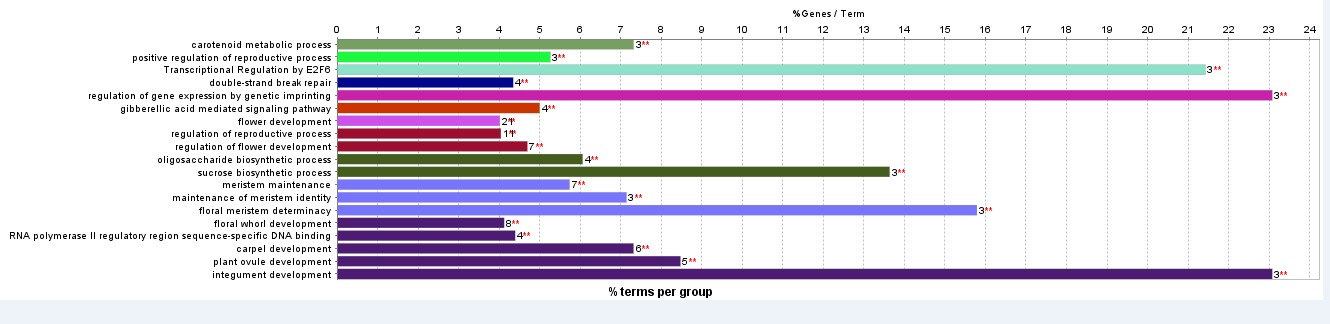


**Fig. S3:Functional characterization of genes in the PA-PPI network identified via Cytoscape – ClueGO^82^ plug-in. Numbers of genes enriched in the constructed PA-PPI network is shown along with their respective biological process.**

**Supplementary Figure S4**

**B) *HK2-BAM1-GRF2-BZR1-AG***

1. ***GA-DELLA (RGA1)-MADs-ARFs***


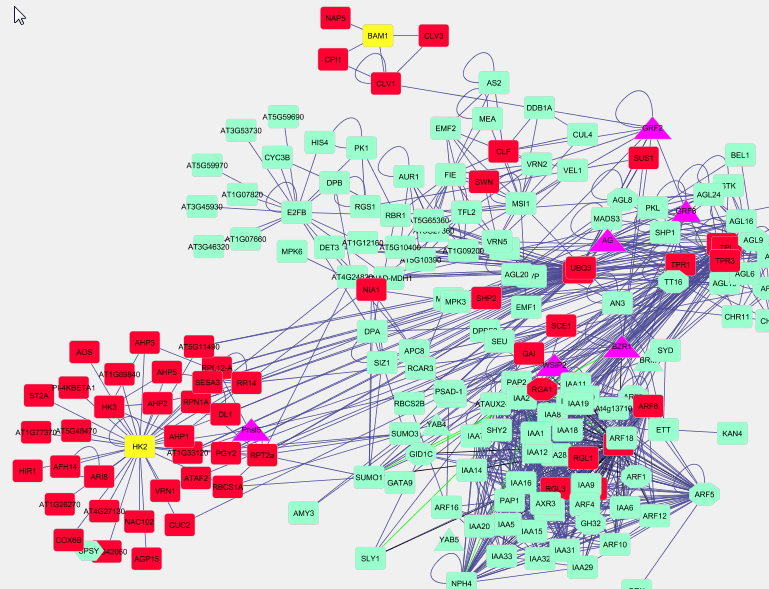

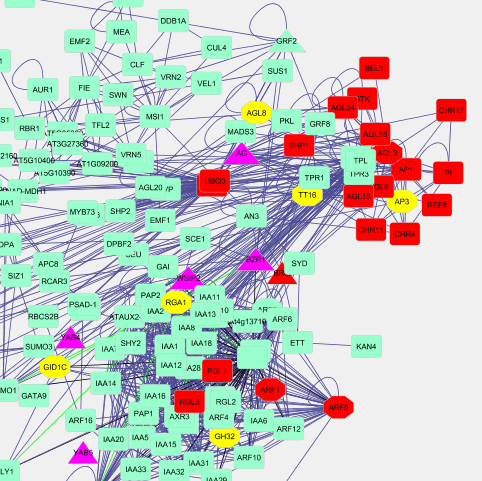


**
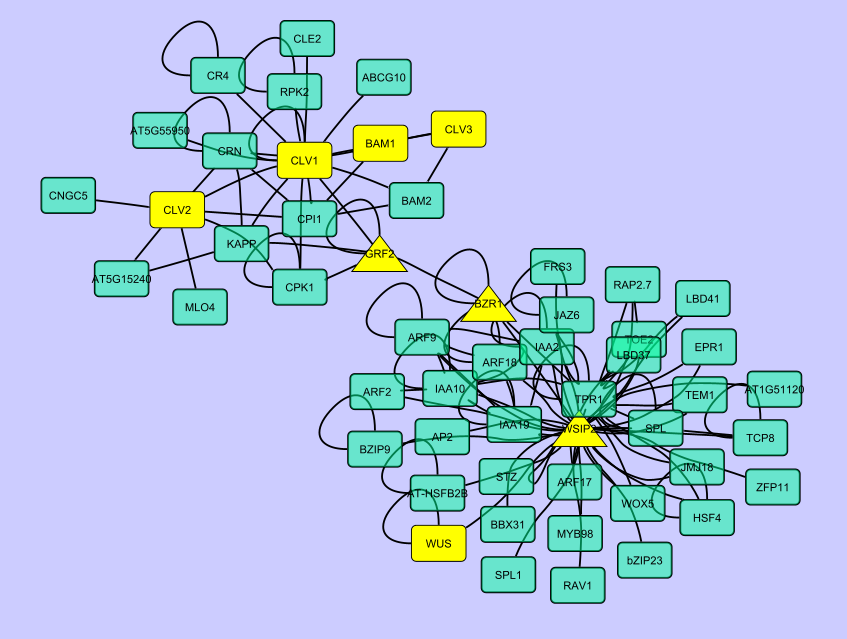
**

**C) *CLV1-WSIP2-GRF2-AG-BZR1***

**Fig. S4: Possible interaction partners of validated genes in the constructed network and their association in hormonal signaling is highlighted using Cytoscape 3.7.1^76,77^. A) Association of *GID1C, RGA1 (DELLA), ARFs, MADS Tfs(AGL8, TT16,and AP3).* B) Association of genes related to *HK2, BAM1, CLV and AG* C) Association of genes related to polycomb proteins.**
