# Supplementary material for: Decoding the molecular mechanism of parthenocarpy in Musa spp. through protein–protein interaction network
Source: Sci Rep. 2021 Jul 16;11:14592. doi: 10.1038/s41598-021-93661-3 (PMC8285514; doi:10.1038/s41598-021-93661-3)
Supplement: Supplementary file 5 — Supplementary Tables. [file 41598_2021_93661_MOESM5_ESM.doc]

***Supplementary Information for***

**Decoding the molecular mechanism of parthenocarpy in *Musa spp*. through protein-protein interaction network**

**Suthanthiram Backiyarani, Rajendran Sasikala, Simeon Sharmiladevi, Subbaraya Uma ***

**ICAR-National Research Centre for Banana, Thogamalai Road, Thayanur Post, Tiruchirapalli 620 102, Tamil Nadu**

*** Correspondence: Uma Subbaraya (**[**umabinit@yahoo.co.in**](mailto:umabinit@yahoo.co.in)**)**

**Telephone No: +91- 0431 2618125**

This Supplementary Information includes information on four tables.

**Supplementary Table S1**

| **Structural properties** | **Value** | **Structural properties** | **Value** |
| --- | --- | --- | --- |
| **Clustering Coefficient** | 0.283 | **Number of Nodes** | 140 |
| **Connected components** | 22 | **Network density** | 0.023 |
| **Network Diameter** | 13 | **Network heterogeneity** | 1.016 |
| **Network Radius** |  | **Isolated Nodes** | 0 |
| **Network centralization** | 0.13 | **Number of self-loops** | 0 |
| **Shortest paths** | 7922(40%) | **Multi-edge node pairs** | 0 |
| **Characteristic path length** | 5.755 | **Analysis time (Sec)** | 42.473 |
| **Avg. number of neighbors** | 3.129 |  |  |

**Table S1.Structural properties of the constructed PA-PPI network in cytoscape^76^**

**Supplementary Table S2**

| **Cluster** | **Score** | **Nodes** | **Edges** | **Node IDs** |
| --- | --- | --- | --- | --- |
| **1** | 10.8 | 11 | 54 | SCL15, BAM1, SCL7, ZEP, GH3.8, EXPA1, At4g13710, RAP23, GAF1, HK2, SL1 |
| **2** | 6 | 7 | 18 | CCA11, SCC13, CKB21, AUR1, SMC3, CTF7, SMC4 |
| **3** | 4.25 | 9 | 17 | SUS1, ODP24, GLGB3, PHSH, ODPA3, ODPA1, GLGS, ODPB3, INV1 |
| **4** | 3.333 | 4 | 5 | GAM1, SPY, D8, GID1C |
| **5** | 3 | 3 | 3 | MAD16, MADS2, AP2 |
| **6** | 3 | 3 | 3 | ARFB, CR15A, ANT |
| **7** | 3 | 3 | 3 | AG, DGAT1, MA653 |
| **8** | **3** | **3** | **3** | A0A1P8AWB4, KSA, KO1 |

**Table S2. Includes details of 8 clusters obtained via MCODE^79^ plugin along with the score, number of nodes and edges in the cluster. Genes in the individual clusters are given under Node IDs.**

**Supplementary Table S3**

| **Pathway** | **No of proteins** | **Proteins** |
| --- | --- | --- |
| **Plant hormone signal transduction** | 50 | IAA14, IAA1, IAA8, IAA13, AHP1, MPK6, IAA16, IAA9, ETT, IAA32, IAA29,HK2, IAA12, RGL2, AHP5, RGA1, IAA18, RR14, RGL3, IAA28, AXR3, SHY2, IAA19, IAA2,IAA7, BZR1, DPBF2, IAA15, ARF1, HK3, IAA6, NPH4, IAA11, MP,GAI, IAA34, PAP2, PAP1, SLY1, GID1C, AHP2, IAA5, RCAR3, RGL1, ATAUX2-11, IAA20, IAA31, IAA30, IAA10, AHP3 |
| **Fatty acid/terpenoid Metabolism** | 5 | GA1, AOS, PI-4KBETA1, ABA1, CPI1 |
| **Ubiquitin mediated proteolysis/Proteosomes** | 7 | CUL4, DDB1A, RPT2a, RPN1A, AT4G24820, SCE1, APC8 |
| **Carbohydrate Metabolism** | 7 | AMY3, SUS1, c-NAD-MDH1, RBCS2B, RBCS1A, AT4G13710 |
| **RNA trasnsprot** | 3 | RPL12-A, AT1G33120, PGY2 |
| **Ribosomes** | 3 | SUMO3,SUMO1,AT4G27130, GAF1 |
| **Lysine Degradation** | 3 | MEA, CLF, SWN |

**Table S3. KEGG^80^ pathway analysis of genes from PA-PPI network predicted to be involved in parthenocarpy.**

**Supplementary Table S4**

| **Gene Name** | **Accession ID^a^** | **Description^b^** | **Primer_F^c^** | **Primer_R^d^** | **Ampliconlength^e^** |
| --- | --- | --- | --- | --- | --- |
| ***MaZEP*** | GSMUA_Achr7P18880_001 | zeaxanthin epoxidase | CTTGCAACTGACGAGGATG | CACCTTGACCCATATTTGGC | 133 |
| ***MaLFY*** | GSMUA_Achr6P16390_001 | floricaula/leafy homolog | GTGGCGAAGAAGGACAGCAA | CCTCTTGGAACGACCTCCTTTTA | 103 |
| ***MaMADS29*** | GSMUA_Achr3P23580_001 | MADS-box transcription factor 29-like | GGATGAGAGACGAGAACGATAAG | CTCGAACCTTGTTGACAGAGTA | 132 |
| ***MaHK2*** | GSMUA_Achr3P22920_001 | histidine kinase CKI1 | AGGGTTTGGAGGTTATTTGG | CATGTCCCTCGGATGTAAAC | 138 |
| ***MaMADS16*** | GSMUA_Achr9P20950_001 | MADS-box transcription factor 16 | GAGAGATGACCACCCAGTTTAC | GAAGGTCATGGGAGCCATATC | 158 |
| ***MaBAM1*** | GSMUA_Achr4P07370_001 | leucine-rich repeat receptor-like serine/threonine-protein kinase | ATGAAAGCGCGGTCTCTAAA | TGATGCCCTGGTACACGATA | 157 |
| ***MaGH3.8*** | GSMUA_Achr4P07220_001 | probable indole-3-acetic acid-amidosynthetase GH3.8 | AGACTGTTGTTCCCGCTAAG | GTTCGTGGTCATCTCCTCTATG | 101 |
| ***MaACLB2*** | GSMUA_Achr11P05030_001 | ATP-citrate synthase beta chain protein 1 | GAGCAGAGACAACAGGGATAAG | CCATCAACATTCAGCACCAAG | 147 |
| ***MaAGL8*** | GSMUA_Achr3P02280_001 | Agamous-like MADS-box protein AGL8 homolog | TCTTCACCGTCCTCATTTCC | GCTTCGCTATCCACTGATCC | 180 |
| ***MaRGA1*** | GSMUA_Achr1P21300_001 | DELLA protein SLR1-like | GGAAAGTGGCCGGGTATT | AGCTCTCGTAGAAGTGTAGGT | 132 |
| ***MaGID1C*** | GSMUA_Achr8P05910_001 | gibberellin receptor GID1C-like | GCTCCTCAACCCGATGTT | CTTCCAATACCAGTCCCTGTC | 103 |
| ***MaEXPA1*** | GSMUA_Achr1P02650_001 | expansin-A1-like | TCCAGTCCATGTCCATCAAAG | GAGAGGGATTGGCTGTTTAGAT | 106 |
| ***MaRPS2*** | GSMUA_Achr5T11130_001 | Putative translational initiation factor 43S | TAGGGATTCCGACGATTTGTTT | TAGCGTCATCATTGGCTGGGA | 84 |

**Table S4. Details of the parthenocarpic candidate genes as a result of PA-PPI network analysis ad their primer sequences used for validation through qRT-PCR**

**^a^ Accession number retrieved from Banana Genome Hub (*Musa* spp.)**

**^b^ Gene description based on homology with *Arabidopsis thaliana* proteins using BLAST2GO^83^**

**^c^&^d^ Forward and reverse primer sequences**

**^e^ Approximate length of the product of semi quantitative PCR.**
